# The preimaginal stages of *Pnigalio gyamiensis* Myartseva & Kurashev, 1990 (Hymenoptera, Eulophidae), a parasitoid associated with *Chrysoesthia sexguttella* (Thunberg) (Lepidoptera, Gelechiidae)

**DOI:** 10.3897/zookeys.214.3266

**Published:** 2012-08-07

**Authors:** Ekaterina Yegorenkova, Zoya Yefremova

**Affiliations:** 1 Department of Geography, Ulyanovsk State Pedagogical University, Ulyanovsk, 432700, Russia; 2 Department of Zoology, The George S. Wise Faculty of Life Sciences, Tel-Aviv University, 69978 Tel-Aviv, Israel

**Keywords:** Chalcidoidea, Eulophidae, *Pnigalio gyamiensis*, *Chrysoesthia sexguttella*, preimaginal morphology, physiological larval functions

## Abstract

The larval instars of *Pnigalio gyamiensis* Myartseva and Kurashev are described in detail for the first time. This species is a larval-pupal ectoparasitoid of *Chrysoesthia sexguttella* (Thunberg) (Lepidoptera, Gelechiidae), which forms leaf mines in the plant *Chenopodium album* L. (Caryophyllales: Amaranthaceae). The female of *Pnigalio gyamiensis* lays a single egg on the skin of the host larva or nearby it, without any significant preference for a particular variant. The presence of long hairs on its body provides the newly-hatched first larval instar with high mobility. Some peculiarities in this parasitoid-host relationship are described.

## Introduction

Five species of the genus *Pnigalio* Schrank were reared from larva of *Chrysoesthia sexguttella* (Noyes 2012): *Pnigalio soemius* (Walker) (Triggiani 1978; Hansson 1987; Rizzo and Massa 2002), *Pnigalio cristatus* Ratzeburg, *Pnigalio incompletus* Bouček, *Pnigalio agraules* Walker (Rizzo and Massa 2002) and *Pnigalio gyamiensis* Myartseva and Kurashev (Myartseva and Kurashev 1990).

Species belonging to *Pnigalio* are ectoparasitoids, with solitary or gregarious larval development; most of them are polyphagous, feeding on several species of leaf miners (Askew 1971, 1984). They attack 70 species from 21 genera of Lepidoptera, some of which are pests of agricultural crops (Schauff et al. 1998). The *Pnigalio* species are potentially important for biological control of lepidopterous leaf miners.

Several species of *Pnigalio* are poorly morphologically characterized and difficult to identify. Consequently, in 2005 Bernardo et al. (2006, 2007) began to study *Pnigalio soemius* species in the laboratory from egg to adult. Bernardo et al. (2008) suggested that *Pnigalio soemius* is a complex of at least two cryptic species, and the same authors (Gebiola et al. 2011) later noted four cryptic species belonging to the *Pnigalio soemius* complex. All the cryptic species are based on morphological, biological, molecular (Bernardo et al. 2008; Gebiola et al. 2010), karyological (Gebiola et al. 2012a) and endosymbiont data (Giorgini et al. 2010). The *Pnigalio soemius* complex is currently considered as a complex of several evolutionary lineages with very little morphological differentiation, but with significant genetic, ecological and biological differences (Gebiola et al. 2012b).

Our reared species is morphologically identical to *Pnigalio gyamiensis*. The DNA sequencing of the *Pnigalio gyamiensis* paratype was analyzed and revealed to be identical to the DNA sequence of *Pnigalio soemius* samples from *Chrysoesthia sexguttella* larvae on *Chenopodium album* and *Atriplex putula*. *Pnigalio gyamiensis* is genetically and biologically well characterized and its taxonomic validity has been confirmed (Gebiola et al. 2012b). Recent literature (Gebiola et al. 2012b) has shown that, based on ITS2 species-specific sequences, the parasitoids reared on the same host-plant system in Italy were genetically identical to the paratype of *Pnigalio gyamiensis* that was described in 1990 by Myartseva and Kurashev and reared on the *Chrysoesthia sexguttella* – *Atriplex* sp. system in Turkmenistan.

The preimaginal stages of *Pnigalio gyamiensis* have never been described. The aim of this work was thus to describe morphologically the preimaginal stages, especially the larval instars; to describe any differences between the physiological functions of each of them; and to elucidate the biological strategies of solitary parasitoids developing inside leaf mines.

## Materials and methods

*Pnigalio gyamiensis* was reared without any other parasitoids from *Chrysoesthia sexguttella* on *Chenopodium album* L. (Caryophyllales: Amaranthaceae). The reared material was studied for the preimaginal stages. Samples of leaf-mines were regularly collected from five different localities in the city of Ul'yanovsk (54°16'N; 48°20'E), (separated from each other by no more than 3 km), between June and mid-September 2009. The food plant of *Chrysoesthia sexguttella* in the Middle Volga region is *Chrysoesthia album*. This plant is widespread, found in fields, gardens, and along roads and paths. *Chrysoesthia sexguttella* has two generations in this area: the first from May to July and the second from August to September.

In total, 500 leaf mines were collected, from which were reared 224 individuals of *Chrysoesthia sexguttella* and 25 specimens of *Pnigalio gyamiensis*. There was only one reared parasitoid of *Pnigalio gyamiensis*. The first generation of *Pnigalio gyamiensis* was reared from mid-June to early July, and the second generation from the end of July to mid-September. Mines from the leaves were stored individually in the laboratory in small glass tubes covered by several layers of wet gauze. In the present study, parasitoids were reared at 25° ± 2°С.

When mines were opened and photographed, this often prevented further development of parasitoid larvae. The total number of observations was 170. The number of observation of each larval instar is given below. In order to assess significant differences we used a non-parametric Fisher's exact test.

Video and photos of larval stages were recorded using a Canon Power Shot A-640. Light microscopy was carried out using a MC-2 ZOOM connected to a digital camera and a Mikromed microscope.

Abbreviation: F1–F4 – length of 1st, 2nd, 3rd and 4th segments of antennal funicle; SMV – submarginal, MV – marginal, PMV – postmarginal and SV – stigmal veins of forewing. Zoological Institute, Russian Academy of Science, St. Petersburg, Russia (ZISP).

## Taxonomic survey

### Pnigalio gyamiensis

Myartseva & Kurashev, 1990

http://species-id.net/wiki/Pnigalio_gyamiensis

Pnigalio gyamiensis Myartseva and Kurashev 1990: 42–43.

#### Morphology.

Our reared specimens were compared with type material (Zoological Institution of Russian Academy of Sciences, St. Petersburg, Russia (ZISP): “Holotype, female, Gami, 3 km W from Ashgabat, ex larva *Chrysoesthia sexguttella* on *Atriplex* sp., 13.10.1986 (Saparmamedova) Myartseva, Kurashev, 1990”, two female paratypes with the same label, and one female with label “Gami, 3 km W from Ashgabat, ex larva *Chrysoesthia sexguttella* on *Atriplex* sp., 30.10.1986 (Saparmamedova) Myartseva, Kurashev, 1990”.

Morphological diagnosis is based on a study of the type material.

Body length 1.08–1.80 mm; F1 1.1–1.3 times as long as F2; F2 1.1–1.2 times as long as F3; F3 1.0 times as short as F4; F4 1.3 times shorter than clava; callus of propodeum with 2 rows of setae: 1 row with 10–12 setae, 2 with 4 setae; sculpture of mesoscutum areolate and size of seta larger than that in scutellum. Forewing 2.3–3.5 times as long as broad; SMV 1.3–1.6 times shorter than MV; MV 1.8–2.7 times longer than PMV; PMV 2.0–3.3 longer than STV; gaster 1.5–1.8 times as long as broad. Body dark blue, the gaster brown with yellow tick at base, legs completely yellow with dark brown last segment of tarsi, hind coxae yellow with brown bracket (in the base of the coxa).

Seventeen females and eight males of *Pnigalio gyamiensis* reared by authors are labelled: **“**Ul'yanovsk, left bank of the river Volga, Verhnaya Terrasa, 56°49'N; 49°44'E, 15 June–8 August 2009 (Yegorenkova)”.

Our species belongs to *Pnigalio gyamiensis* and its morphological variability is less high.

#### Female.

Body length 1.35–1.80 mm; F1 1.2–1.3 times as long as F2; F2 1.1 times as long as F3; F3 1.0–1.1 times as short as F4; F4 1.3–1.4 times shorter than clava; forewing 2.5–2.8 times as long as broad; SMV 1.5–1.6 times shorter than MV; MV 1.7–1.8 times longer than PMV; PMV 2.7–2.8 longer than STV; gaster 1.4–1.5 times as long as broad. Body dark green with metallic tint, gaster with yellow tick or spot in the base of the gaster, legs completely yellow with dark brown last segment of tarsi, hind coxae mostly yellow without brown bracket. **Male** (first description): body length 1.25–1.38 mm; thorax 1.6 times as long as broad; pronotum 1.7 times as broad as long; sculpture of scutellum is the same as that of the mesoscutum; propodeum 2.3 times as broad as long; gaster 1.7–1.8 times as long as broad. Colouring is the same as in the female, sometimes hind femur and fourth tarsal segments darkened**.**

#### Distribution.

Turkmenistan (Myartseva, Kurashev, 1990), Italy (Gebiola et al. 2012b). New record for Middle Volga Basin (Russia).

#### Biology.

Larval solitary ectoparasitoid.

### Description of preimaginal stages of *Pnigalio gyamiensis*

**Egg**

**Figure 1–2.**
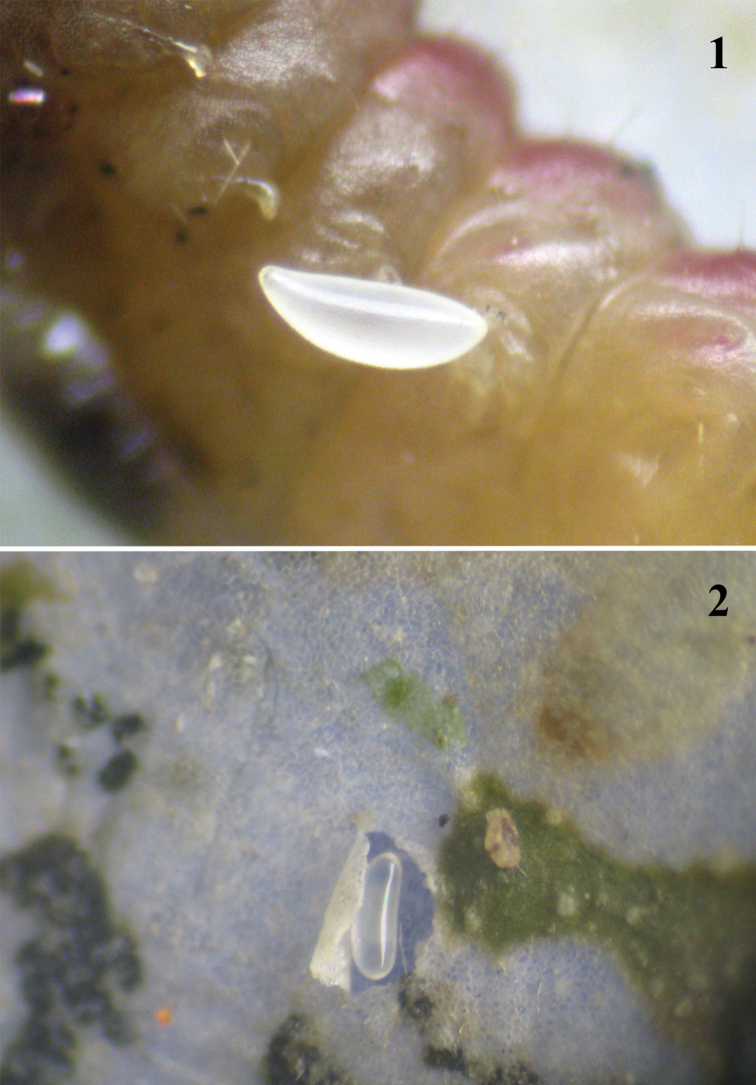
**1** A recently laid egg of the female of *Pnigalio gyamiensis*, on the IV–V segments of the second larval instar of *Chrysoesthia sexguttella* (ventral view) **2** *Pnigalio gyamiensis* egg with developing embryo in an opened mine of *Chrysoesthia sexguttella* beside the host remains.

**Figure 3–4:**
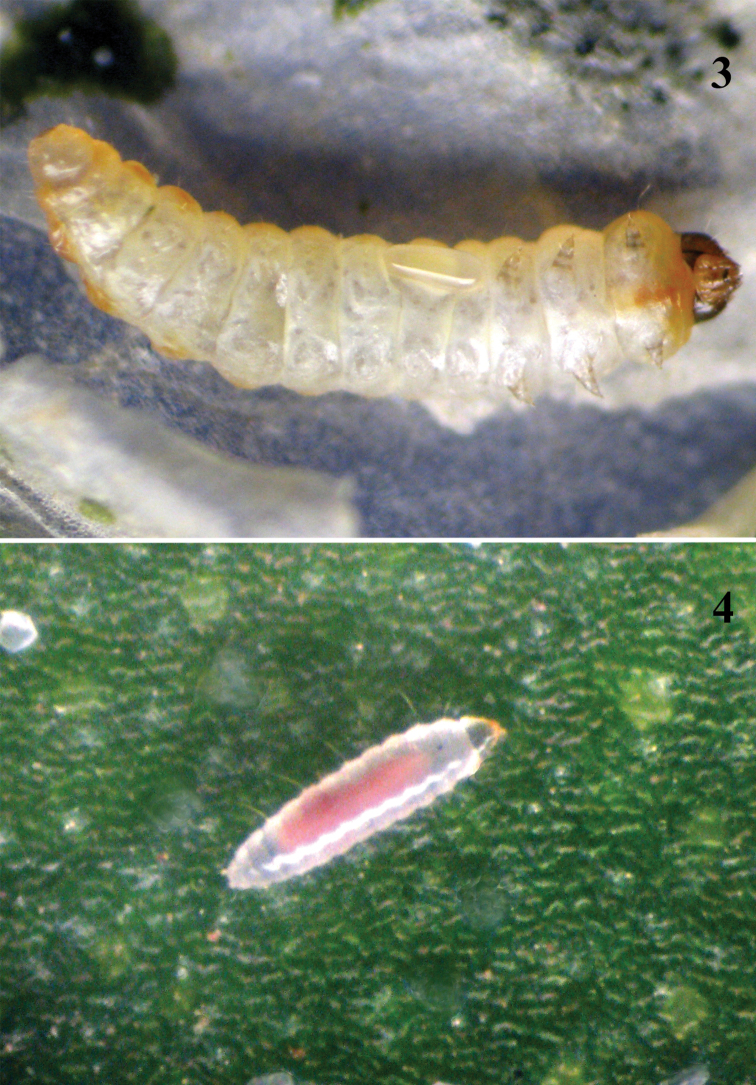
**. 3** *Pnigalio gyamiensis* egg with developing embryo on IV–V segments of *Chrysoesthia sexguttella* larva (ventral view) **4** 1^st^ instar *Pnigalio gyamiensis* larva in the mine of *Chrysoesthia sexguttella*

The shape of the egg changes during development of the embryo. The egg (Figure 1) just laid by a female of the parasitoid is oblong, both ends are rounded, with one a little broader than the other. The egg is white and shiny without sculpture. As the embryo develops, the egg becomes oval. Such eggs were either found beside the host (larva of dead *Chrysoesthia sexguttella*) (Figure 2) or lying on the surface of the host's cuticle. An egg with a fully developed embryo is elongate (Figure 3).

Eggs of species of the genus *Pnigalio* were previously studied by Delenoue and Arambourg (1967) and Gebiola et al. (2009). The *Pnigalio soemius* embryo can also develop successfully within its egg without a host. In 17 cases the egg was found near the host, and in 20 cases on the skin of the host (n=37). The differences are not significant (Fisher's exact test: p>0.05). Development of the egg lasts on average 2.3 ± 0.8 days before hatching of the first larval instar. It is possible that a newly-hatched first larval instar reaches the host using the long hairs on its body to facilitate movement inside the leaf mine (when the egg is laid near to the host).

**1^st^ instar larva**

*Morphology*. The larva has 13 distinct segments including the extended head, which is 1.3 times as long as the second thoracic segment. The head capsule is dark yellow with one brown mandibular tooth that is used to puncture the cuticle of the host (Figure 4). The shape of the mandible is triangular. Chaetotaxy: the head capsule is covered by hairs. The body has 2 lateral rows of protuberances on segments II, III, IV, VI, VIII, X, XII and XIII, each with long hairs (total 32 hairs). Length of the hairs is equal to the diameter of the abdominal segments. Long hairs are at an angle of 45° to the body and at an obtuse angle to each other. The first dorsal hairs bend towards the head while the last ones bend back towards the anus.

*Behaviour*. The larva is very active and quickly moves in the mine by means of muscle contractions, which are clearly visible. The parasitoid larva may feed on the haemolymph but does not do so solely. During observation of this instar it was noted that the larva punctured (drilled into) the cuticle of the host anticlockwise, thereby gaining access to the haemolymph. We observed such larva externally on the host larva and in a mine without a host, where it was probably searching for a host. At the end of this instar the larva becomes less active and moults to the second instar on the surface of the host's body (33 observations).This stage lasts on average 3.8 ± 0.7 days.

**2^nd^ instar larva**

**Figure 5–6.**
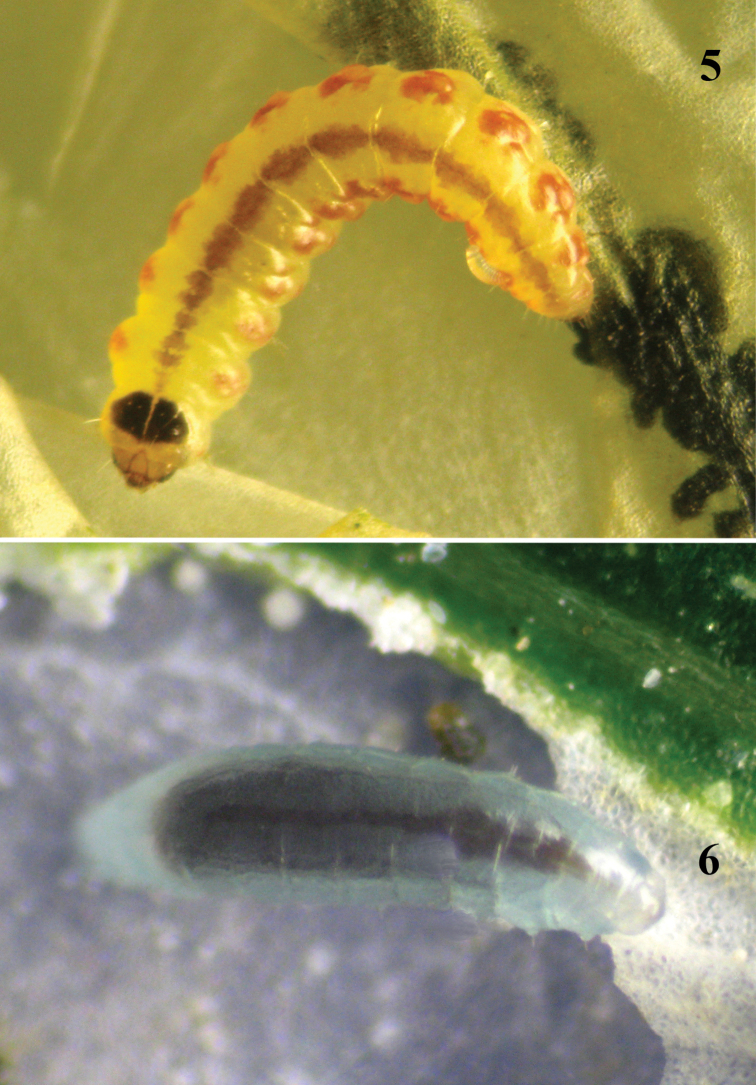
Larva of *Chrysoesthia sexguttella* (dorsal view) parasitized by 3^rd^ instar larva of *Pnigalio gyamiensis* **6** 3^rd^ instar *Pnigalio gyamiensis* larva (protuberances are visible, ventral view).

*Morphology*. The second instar larva is larger than the first, less active, and the body is segmented (Figure 5). Pulsation of the gut becomes distinct, and the food (firstly pale yellow and later on darkened) is moved to the anal part of gut. This larva moults to the 3^rd^ instar on the surface of the host and the larva's head loses any distinctive shape as well as its hairs.

**Figure 7–8.**
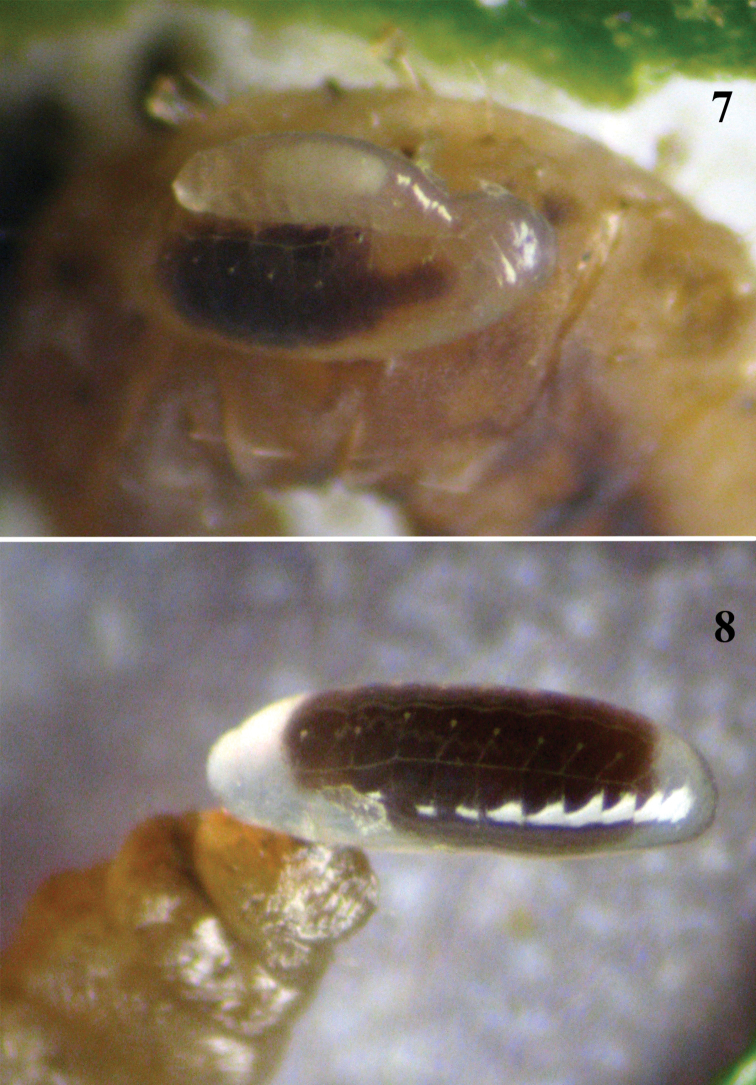
**7** Siblicide behavior exhibited by 2^nd^ and 4^th^ larval instars of *Pnigalio gyamiensis* on a larva of *Chrysoesthia sexguttella* (dorsal view) **8** 4^th^ larval instar of *Pnigalio gyamiensis* on a larva of *Chrysoesthia sexguttella* inside its mine.

*Behaviour*. In contrast to larva of the 1^st^ instar that leaves the host several times, this 2^nd^ larva stays on the host, feeding almost entirely on the haemolymph. The 2^nd^ larval instar spends much longer periods feeding than that of the 1^st^ instar larva, resulting in a rapid increase in size. We observed siblicide behavior between the 2^nd^ larval and 4^th^ larval instar of *Pnigalio soemius* (Figure 7). It means the 2^nd^ larval instar begins to feed on the haemolymph of 4^th^ instar larva (28 observations).This stage lasts on average 2.9 ± 0.6 days.

**3^rd^ instar larva**

*Morphology*. The 3^rd^ instar larva (Figure 6) has distinct protuberances in some segments (II–IV thoracic, VI, VIII, X and XII abdominal, and XIII anal). Length of a protuberance is equal to its width. Each protuberance has hair 1.5 times as long as length of protuberance.

*Behaviour*. The larva is actively feeding at this stage and its gut reveals a visible pulsation. We did not observed siblicide behavior between two larvae of 3^rd^ instar. This stage lasts on average 2.5 ± 0.7 days (20 observations).

**4^th^ instar larva / prepupa**

*Morphology*. The 4^th^ larval instar (Figure 8) lacks mobility and has nine visible pairs of spiracles of the respiratory system on the II and III thoracic and I–VII abdominal segments. The fully fed larva has a dark brown gut that pulsates in one direction for 40 seconds and then in the opposite direction for 40 seconds. It is important to note that as the larva develops the frequency of pulsation decreases to a rate of 60 seconds in one direction and 60 seconds back. The prepupa loses segmentation and the body instead forms two sections between head and thorax and thorax and abdomen.

**Figure 9–10.**
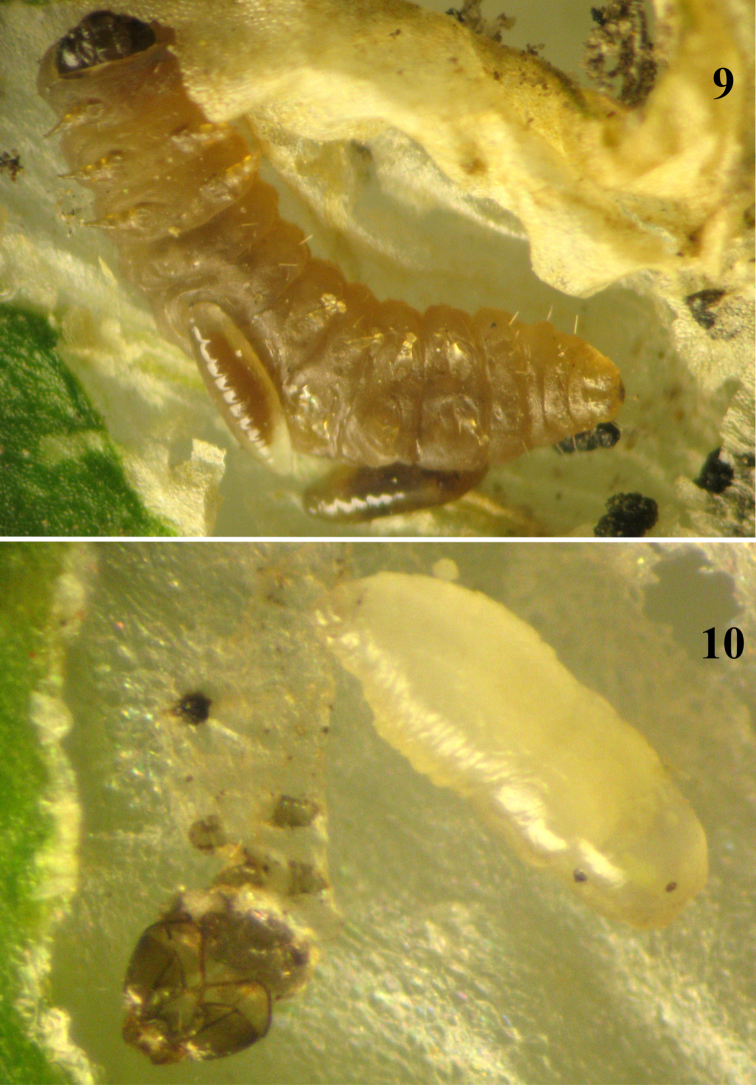
**9** Two 4^th^ larval instar *Pnigalio gyamiensis* on a larva of *Chrysoesthia sexguttella* inside its mine **10** Prepupa of *Pnigalio gyamiensis* in an opened mine of *Chrysoesthia sexguttella*.

*Behaviour*. At the end of this stage the parasitoid larva leaves the host, stops feeding and loses mobility; its gut is full and equal to 75% of body weight. Antagonistic behavior by larvae against larvae of the same species was not observed; larvae of 4^th^ instar may feed on the same host independently of each other (Figure 9) but might be attack by larvae of the 2^nd^ instar (see Figure 7). This stage lasts on average 2.9 ± 0.6 days (24 observations).

**Pupa**

**Figure 11–12.**
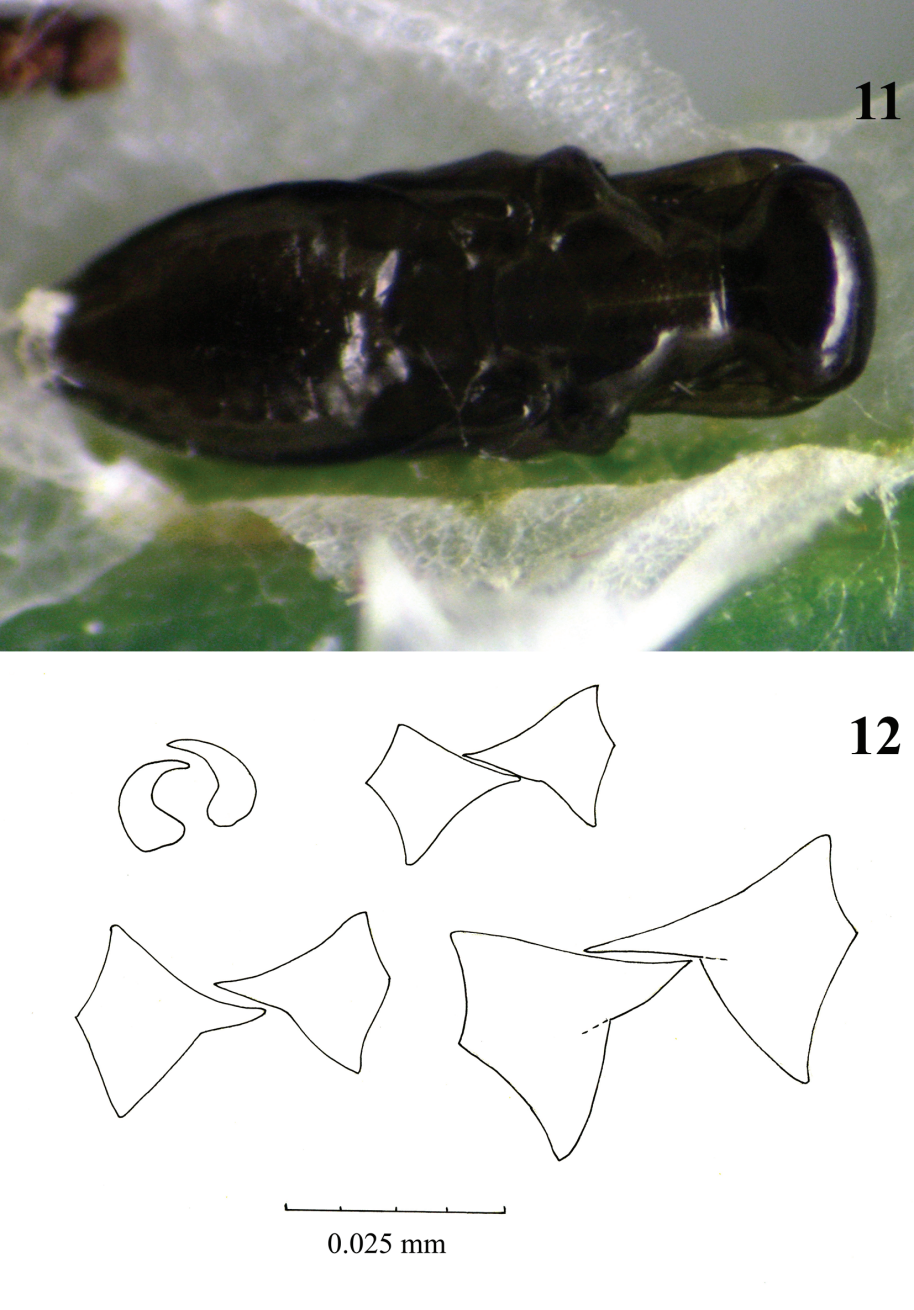
**11** Fully developed pupa of *Pnigalio gyamiensis* in a mine of *Chrysoesthia sexguttella* (dorsal view) **12** Mandibles of larval instars of *Pnigalio gyamiensis*: top row mandibles of 1st and 2nd larval instars, bottom row mandibles of 3rd and 4th larval instars.

*Morphology*. The pupa attaches to the leaf epidermis (Figure 10).The pupa is initially white or slightly yellow and then begins to darken to dark brown or black. The fully developed pupa of *Pnigalio gyamiensis* (Figure 11) has a metallic tint but the adult is never visible through the chitinized exuviae of pupa. Female pupa is recognizable by their large gaster and ovipositor visible through the light coloured cuticle of the gaster in the early stage of pupation, whereas the male pupa has a smaller gaster and darker colour.

The pupa is situated inside the mine ventrally to the leaf's surface. It develops on average 5.4 ± 0.8 days (28 observations).

The total duration of development is 19.8 ± 1.2 days.

*Behaviour*. The adult exits through the oral cavity of the pupa often in the early morning. The adult begins to clean its antennae and head and then leaves the mine.

## Conclusion

*Pnigalio gyamiensis* presents four larval instars and the three moults are easy recognizable. The 1^st^ instar larva is clearly visibly by the presence of long hairs on its body. The mandibles are very small and curved, and used to hook onto the cuticle of the host (Figure 12). Some authors have noted a difficulty in differentiating larval instars, such as in *Hyssopus pallidus* Askew (Tschudi-Rein and Dorn 2001), with the only discernible differences being in the shape and size of the mandibles. They did not report the long hairs on the body that the larva uses for moving across the surface of the host or inside the mine. The 2^nd^ larval instar loses these long hairs and moves slowly; it is recognizable by its mandibles. The mandibles of 2^nd^, 3^rd^ and 4^th^ instar larvae (Figure 12) differ in size but the 4^th^ instar has one large, well-development tooth.

**Figure 13–14.**
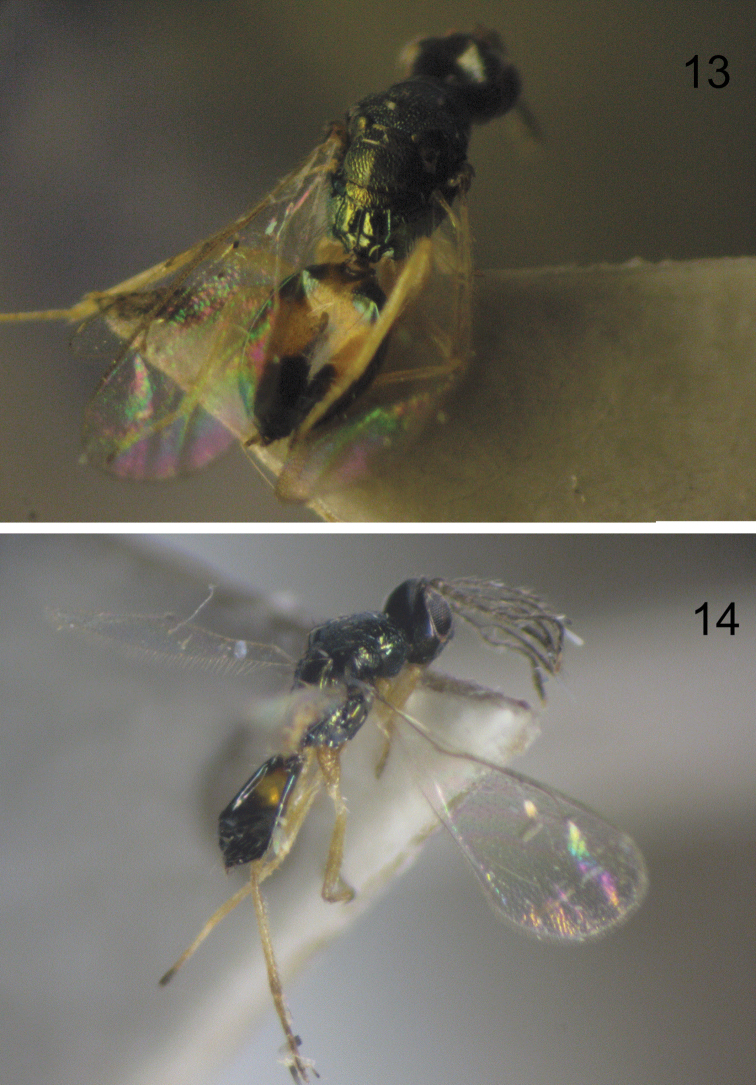
**13** Emerged female of *Pnigalio gyamiensis* **14** Emerged male of *Pnigalio gyamiensis*.

The emerged adults (both sexes) are shown in Figure 13, 14. The female parasitoid *Pnigalio gyamiensis* paralyses the larva *Chrysoesthia sexguttella*, which loses mobility, stops feeding and dies. The parasitoid larva then feeds on the killed host. Only a few cases were observed of the parasitoid female having laid an egg on the skin of a dead host larva; but in each case the parasitoid larva developed successfully. The female hid her egg on the skin of the host larva or near it without significant preference for any of the variants. The high mobility of the 1st instar allows the larva to find a host quickly and begin to feed.

## Supplementary Material

XML Treatment for Pnigalio gyamiensis

## References

[B1] Askew RR (1971) Parasitic insects. American Elsevier Publishing Co., Inc., New York, xvii + 316 pp.

[B2] Askew RR (1984) Species of *Pnigalio* and *Chrysocharis* (Hymenoptera: Eulophidae) parasitic on Tischeriidae (Lepidoptera), with the description of a new species. Entomologist's Gazette 25: 103-109.

[B3] Bernardo U Pedata PA Viggiani G (2006) Life history of *Pnigalio soemius* (Walker) (Hymenoptera: Eulophidae) and its impact on a leafminer host through parasitization, destructive host-feeding and host-stinging behavior. Biological Control 37: 98-107. doi: 10.1016/j.biocontrol.2005.11.011

[B4] Bernardo U Pedata PA Viggiani G (2007) Phenotypic plasticity of pigmentation and morphometrical traits in *Pnigalio soemius* (Walker) (Hymenoptera: Eulophidae). Bulletin of Entomological Research 97 (1): 101-109. doi: 10.1017/S0007485307004816 17298687

[B5] Bernardo U Monti MM Nappo AG Gebiola M Russo A Pedata PA Viggiani G (2008) Species status of two populations of *Pnigalio soemius* (Hymenoptera: Eulophidae) reared from two different hosts: An integrative approach. Biological Control 46: 293-303. doi: 10.1016/j.biocontrol.2008.05.009

[B6] Delenoue CP Arambourg Y (1967) Contribution à l'étude en laboratoire de *Pnigalio mediterraneus* (Hymenoptera: Chalcidoidea, Eulophidae). Annales de la Société Entomologique de France 3: 909-927.

[B7] Gebiola M Bernardo U Monti MM Navone P Viggiani G (2009) *Pnigalio agraules* (Walker) and *Pnigalio mediterraneus* Ferrière and Delucchi (Hymenoptera: Eulophidae): two closely related valid species. Journal of Natural History 43 (39): 2465-2480. doi: 10.1080/00222930903105088

[B8] Gebiola M Bernardo U Burks RA (2010) A reevaluation of the generic limits of *Pnigalio* Schrank (Hymenoptera: Eulophidae) based on molecular and morphological evidence. Zootaxa 2484: 35-44.

[B9] Gebiola MM Giorgini, Navone P Bernardo U (2012a) A karyological study of the genus *Pnigalio* Schrank (Hymenoptera: Eulophidae): Assessing the taxonomic utility of chromosomes at the species level. Bulletin of Entomological Research 102: 43-50. doi: 10.1017/S0007485311000356 21736855

[B10] Gebiola M Gómez-ZuritaJ, Monter MM Navone P Bernardo U (2012b) Integration of molecular, ecological, morphological and endosymbiont data for species delimitation within the *Pnigalio soemius* complex (Hymenoptera: Eulophidae). Molecular Ecology 1–18. 10.1111/j.1365-294X.2011.05428.x 22268975

[B11] Giorgini M Bernardo U Monti MM Nappo AG Gebiola M (2010) *Rickettsia* Symbionts Cause Partheno genetic Reproduction in the Parasitoid Wasp *Pnigalio soemius* (Hymenoptera: Eulophidae). Applied and Environmental Microbiology 76 (8): 2589-2599. doi: 10.1128/AEM.03154-09 20173065 PMC2849191

[B12] Hansson C (1987) New records of Swedish Eulophidae and Pteromalidae (Hymenoptera: Chalcidoidea), with data on host species. Entomologisk Tidskrift 108 (4): 167-173.

[B13] Myartseva SN Kurashev VN (1990) Noviye vidi roda *Pnigalio* Schrank (Hymenoptera, Eulophidae) – entomofagi miniruyushchikh nasekomikh v prikopetdagskoy zonye Turkmenii. (New species of *Pnigalio Schrank* (Hymenoptera: Eulophidae) – entomophages of mining insects in Kopetdag zone of Turkmenia. ) Izvestiya Akademii Nauk Turkmenskoy SSR (Seriya Biologicheskikh Nauk) 1990 (2): 42-43 [in Russian]

[B14] Noyes JS (2012) Universal Chalcidoidea Database, World Wide Web electronic publication. The Natural History Museum, London. http://ww.nhm.ac.uk/entomology/chalcidoids /index. html [access on 6 July 2012]

[B15] Rizzo MC Massa B (2002) Ecology of the eulophid parasitoid community living on hosts of spontaneous flora linked to citrus grove (Hymenoptera: Chalcidoidea: Eulophidae). In: Melika G, Thuryczy C (Eds) Parasitic wasps: evolution, systematics, biodiversity and biological control. International symposium: “Parasitic Hymenoptera: Taxonomy and Biological Control” (14–17 May 2001, Kőszeg, Hungary). Agroinform Kiady and Nyomda, Budapest, Hungary, 357 pp.

[B16] Schauff ME LaSalle J Wijesekara GA (1998) The genera of chalcid parasitoids (Hymenoptera: Chalcidoidea) of citrus leafminer *Phyllocnistis citrella* Stainton (Lepidoptera: Gracillariidae). Journal of Natural History 32: 1001-1056. doi: 10.1080/00222939800770521

[B17] Triggiani O (1978) *Microsetia sexguttella* Thunberg (Lepidoptera: Gelechiidae) a member of the microlepidoptera mining the leaves of *Chenopodium album*. Entomologica, Bari 14: 9-24.

[B18] Tschudi-Pein K Dorn S (2001) Reproduction and immature development of *Hyssopus pallidus* (Hymenoptera: Eulophidae), an ectoparasitoid of the codling moth. European Journal of Entomology 98: 41-45.

